# Study on Appropriate Rectal Volume for External Irradiation in Patients With Cervical Cancer

**DOI:** 10.3389/fonc.2022.814414

**Published:** 2022-02-22

**Authors:** Yanjiao Wu, Chunmei Liu, Wenyan Wang, Lei Tian, Zhiqing Xiao, Yanqiang Wang, Han Guo, Xiaoying Xue

**Affiliations:** Department of Radiotherapy, The Second Hospital of Hebei Medical University, Shijiazhuang, China

**Keywords:** cervical cancer, rectum volume, external pelvic irradiation, organ at risk, radiation proctitis

## Abstract

**Objective:**

To investigate the appropriate rectal volume for external irradiation of cervical cancer.

**Methods:**

A retrospective study of 143 patients with cervical cancer who underwent external radiotherapy from January 2017 to September 2020 was conducted. Average rectum volumes and the cumulative dose (V30, V40, V50, D2cc) to organs at risk (bladder, rectum, and small bowel) during radiotherapy were evaluated using the treatment planning system. Rates of radiation cystitis and radiation proctitis were assessed.

**Results:**

The median follow-up was 48 months, and the included patients had a median age of 53 years. Patients were divided into 3 groups based on their average rectum volume: Group A: <40 ml; Group B: 40–70 ml; and Group C: ≥70 ml. V30 and V40 in the rectum bladder and small bowel were highest in Group A (mean ± SD standard deviation), but V50 and D2cc in the rectum and bladder were highest in Group C (mean ± SD). Patients in Group B had the lower incidence of both radiation cystitis and radiation proctitis. (p<0.05).

**Conclusions:**

For external irradiation in patients with cervical cancer, a rectum volume of 40–70 ml seems most appropriate, whereas >70 ml increases the risk of severe radiation cystitis and radiation proctitis, and <40 ml increases the risk of mild radiation cystitis and mild radiation proctitis.

## Background

Cervical cancer is one of the most common gynecological malignancies, ranking the third most common cancer among women worldwide. According to epidemiological data, there are about 500,000 new cases in the world every year, accounting for 5% of all new cancer patients. Among them, 85% of cervical cancer cases occur in developing countries, and its mortality rate ranks second among female tumors (1).

The standard treatment for patients with locally advanced cervical cancer is now a combined modality approach (chemotherapy and radiation therapy); the radiation therapy consists of external beam irradiation, followed by brachytherapy to boost the gross tumor in the cervix (2). The use of intensity-modulated radiation therapy (IMRT) and spiral tomography (TOMO) has significant advantages for radiotherapy of cervical cancer, effectively reducing the dose to surrounding organs while ensuring that the target area is covered to meet clinical requirements (3–7). Mounting clinical evidence correlates tumor control rate with dose. For instance, a retrospective study has shown that high-risk clinical target volume (CTVHR) dose -85 Gy (EDQ2, D90) delivered in 7 weeks provides a 3-year local control rate >94% in limited-size (20 cm^3^), >93% in intermediate-size (30 cm^3^), and >86% in large-size CTVHR (70 cm^3^) tumors (8). However, alarmingly high gastrointestinal (35% Grade 3 and 4) and genitourinary (9% Grade 3 and 4) complications were documented in patients who received chemo-irradiation in the published RTOG 90-01 and 92-10 studies (9–11). The incidence of radiation cystitis and radiation proctitis is related not only to the patient’s physical condition but also to the dose received (12).

As a relatively controllable influencing factor, rectum volume not only changes the dose of radiotherapy but also provides a new idea for the prevention of radiotherapy complications. We have a patent for a rectal balloon used in prostate cancer radiotherapy, patent number: CN 201320826810, which can fix the position and volume of rectum. Therefore, we conducted this study to find the right rectal volume for external irradiation of cervical cancer.

## Materials

This clinical trial was approved by the Ethics Committee of the second hospital of Hebei Medical University(2021-P041). The trial was performed in accordance with the standards for human clinical trials and the principles stated in the Declaration of Helsinki. All patients signed an informed consent form before enrollment in this study.

### Patients

This study retrospectively included patients who received external radiotherapy for cervical cancer from January 2017 to September 2020. The inclusion criteria were as follows: 1. pathological diagnosis of squamous cell carcinoma cervical cancer; 2. no prior pelvic radiotherapy; 3. FIGO staging was completed before radiotherapy; 4. KPS (Karnofsky score) ≥ 70; 5. no history of other malignant comorbidities; 6. no contraindication to CT/MRI scanning and history of corresponding contrast allergy; 7. willing to actively cooperate with the completion of the study project and follow-up requirements; and 8. signed informed consent. The exclusion criteria were as follows: 1. non-squamous cell cervical lesions; 2. previous pelvic radiotherapy; 3. dropping out of treatment before completion; 4. KPS <70; 5. history of other tumors; 6. contraindication to CT/MRI scan and history of allergy to the corresponding contrast agent without enhancement scan; and 7. refused to sign informed consent.

### External Radiation Therapy

The rectum and bladder were emptied 1 h before the CT scan, and 500 ml of purified water was immediately consumed. The patient was placed in a supine position with the thermoplastic body film in a fixed position. Enhanced CT localization was performed using a Philips large aperture CT simulator; two senior radiotherapy doctors outlined the target area with CT and MR fusion and specified the radiotherapy plan. All patients were treated with external pelvic irradiation combined with intracavitary brachytherapy and simultaneous platinum-based chemotherapy. Pelvic external irradiation was performed with IMRT (simultaneous integrated boost intensity-modulated radiation therapy) or TOMO (helical tomographic radiotherapy). As recommended by National Comprehensive Cancer Network guidelines, external beam radiation therapy applies 45–50 Gy/25 fractions once a day, 5 times a week. Following HDR brachytherapy, 6 Gy/5 fractions or 7 Gy/4 fractions were applied for patients in the radical radiotherapy group; 5.5 Gy/2 fractions or 6 Gy/3 fractions were applied for the postoperative radiotherapy group.

The standard outline of organs at risk was as follows: 1) bladder outline—outline in the filled state, from the fornix upward to the base; 2) rectum outline—upper border, migrate to the sigmoid colon, usually at the S2-3 gap; the lower boundary is the anal margin marked by lead dots during simulated positioning; 3) small bowel outline—refers to RTOG guidelines for the normal pelvic tissue outline, the upper border is the lower border of the pubic symphysis or the anal orifice, with the lower border 3–44 cm above the inferior border of the pubic symphysis or the anal orifice; 4) small bowel outline—referring to the RTOG guidelines for outlining normal pelvic tissues, the bowel-bag outline method was used: the upper border was 1 cm above the PTV, and the lower border was the lowermost layer of the intestinal canal, outlining all abdominal contents except the abdominal wall muscle and bone tissue and removing non-intestinal normal tissues such as the bladder. The dose and volume limits of organs at risk in the pelvic cavity are as follows: rectum V40 < 40%, bladder V40 < 40%, small intestine V40 < 40%, bone marrow V10 < 90%, femoral head V50< 5%, kidney V33 < 33%, spinal cord 0.1 cc<45 Gy.

Some cases meeting the inclusion criteria were recorded on the planning system showing data as follows: rectum volume, rectum V30, rectum V40, rectum V50, rectum D2cc; bladder V30, bladder V40, bladder V50, bladder D2cc; small bowel V30, small bowel V40, small bowel V50, small bowel D2cc.

These patients were divided into three groups based on different rectum volumes: Group A: <40 ml; Group B: 40–70 ml; and Group C: ≥70 ml. The different rectum volumes and cumulative doses to the rectum (V30, V40, V50, D2cc), bladder (V30, V40, V50, D2cc), and small intestine (V30, V40, V50, D2cc) were calculated using the treatment planning system.

All patients were followed up and graded according to radiocystitis and radiation proctitis grading criteria (13, 14), and the incidence of radiocystitis and radiation proctitis in each group was tallied.

### Statistical Methods

All data analyses were performed using SPSS 26.0 (SPSS Inc, Chicago, IL, USA). Baseline characteristics such as age, FIGO stage, pelvic LN metastasis, and rectum volume were compared between dose groups by the t-test or X^2^ test. Doses to organs at risk were compared using non-linear analysis and t-tests, and odds of developing radiation cystitis and radiation proctitis were compared using the X^2^ test. A p value less than 0.05 was considered statistically significant for all tests.

## Results

### Patient Baseline Characteristics

A total of 160 patients with cervical cancer met the criteria for inclusion. Based on the exclusion criteria, 17 patients were excluded, resulting in 143 patients being included in the study. There were 10 patients lost to follow-up, and 10 patients had died; 143 patients were finally followed up. The median age of the patients was 53 years, ranging from 25 to 77 years. 3 patients were in stage IA, 27 in stage IB, 28 in stage IIA, 32 in stage IIB, 5 in stage IIIA, 7 in stage IIIB, 34 in stage IIIC, 6 in stage IVA, and 1 in stage IVB. TOMO radiotherapy was used in 10 cases and IMRT radiotherapy in 133. The 143 patients were divided into three groups based on different rectum volumes: Group A: <40 ml; Group B: 40–70 ml; and Group C: ≥70 ml. The comparison of baseline characteristics between the groups is shown in **Table 1**. Baseline characteristics such as age, KPS score, FIGO stage, and pelvic LN metastasis did not differ significantly between the groups (**Table 1**).

**Table 1:** Clinical characteristics of patients.

Characteristic	Group A (n = 21)	Group B (n = 59)	Group C (n = 63)	p
Age (years), mean	51.57 ± 10.38	53.83 ± 9.853	52.51 ± 10.53	0.6279
FIGO stage (n)				0.9476
IA	1	2	0	
IB	3	12	12	
IIA	3	11	14	
IIB	3	15	14	
IIIA	1	2	2	
IIIB	2	2	3	
IIIC	7	12	15	
IVA	1	3	2	
IVB	0	0	1	
Pelvic LN metastasis (n)				0.5443
YES	8	15	18	
NO	13	44	45	
Rectum volume (cm^3^), mean	32.90 ± 4.908	53.68 ± 8.984	111.9 ± 40.23	<0.0001

### Analysis of Cumulative Dose to Organs at Risk

**Figure 1:**
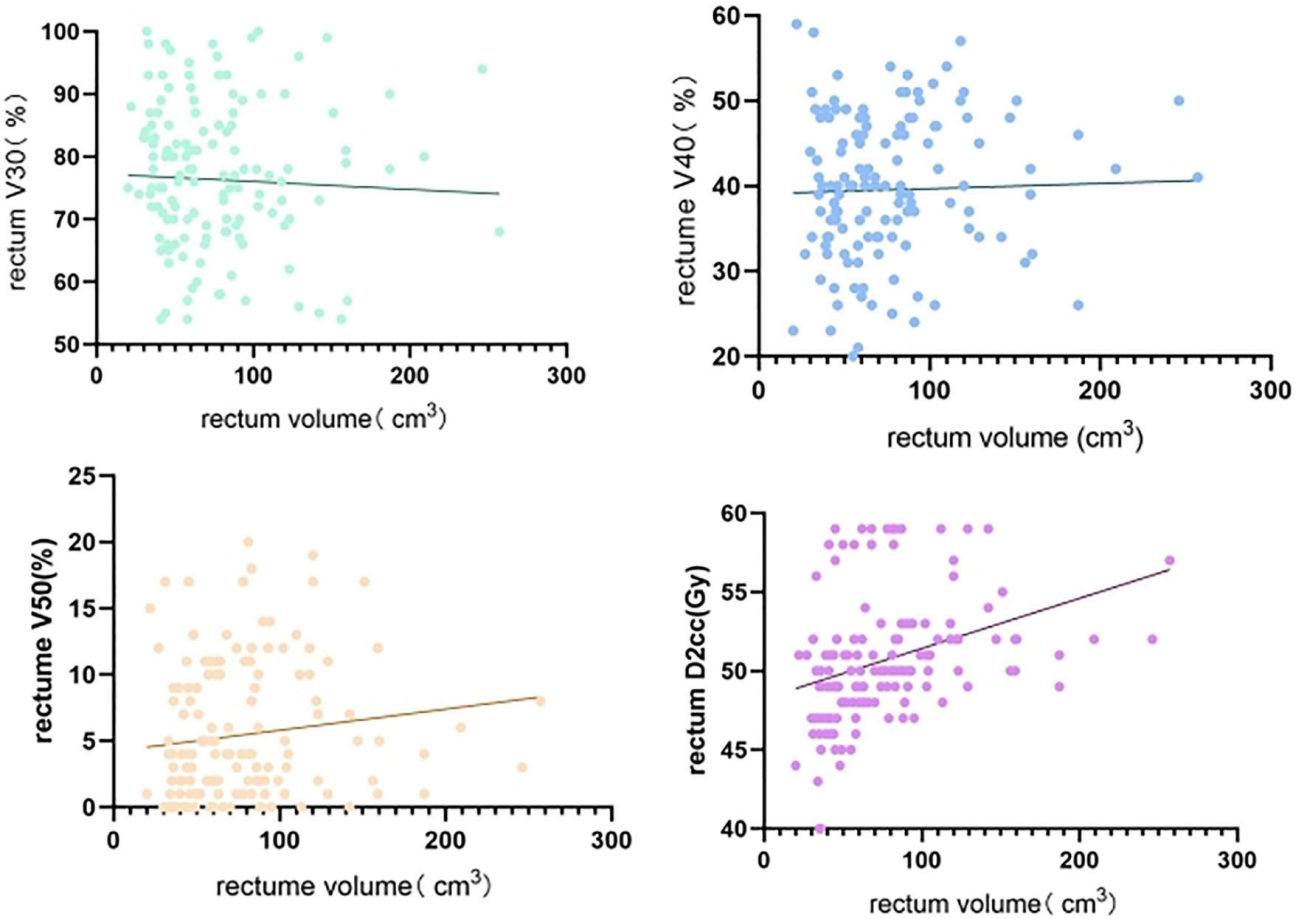
Non-linear regression analysis of rectum volume increase on rectum V30, V40, V50, D2cc.

**Figure 2:**
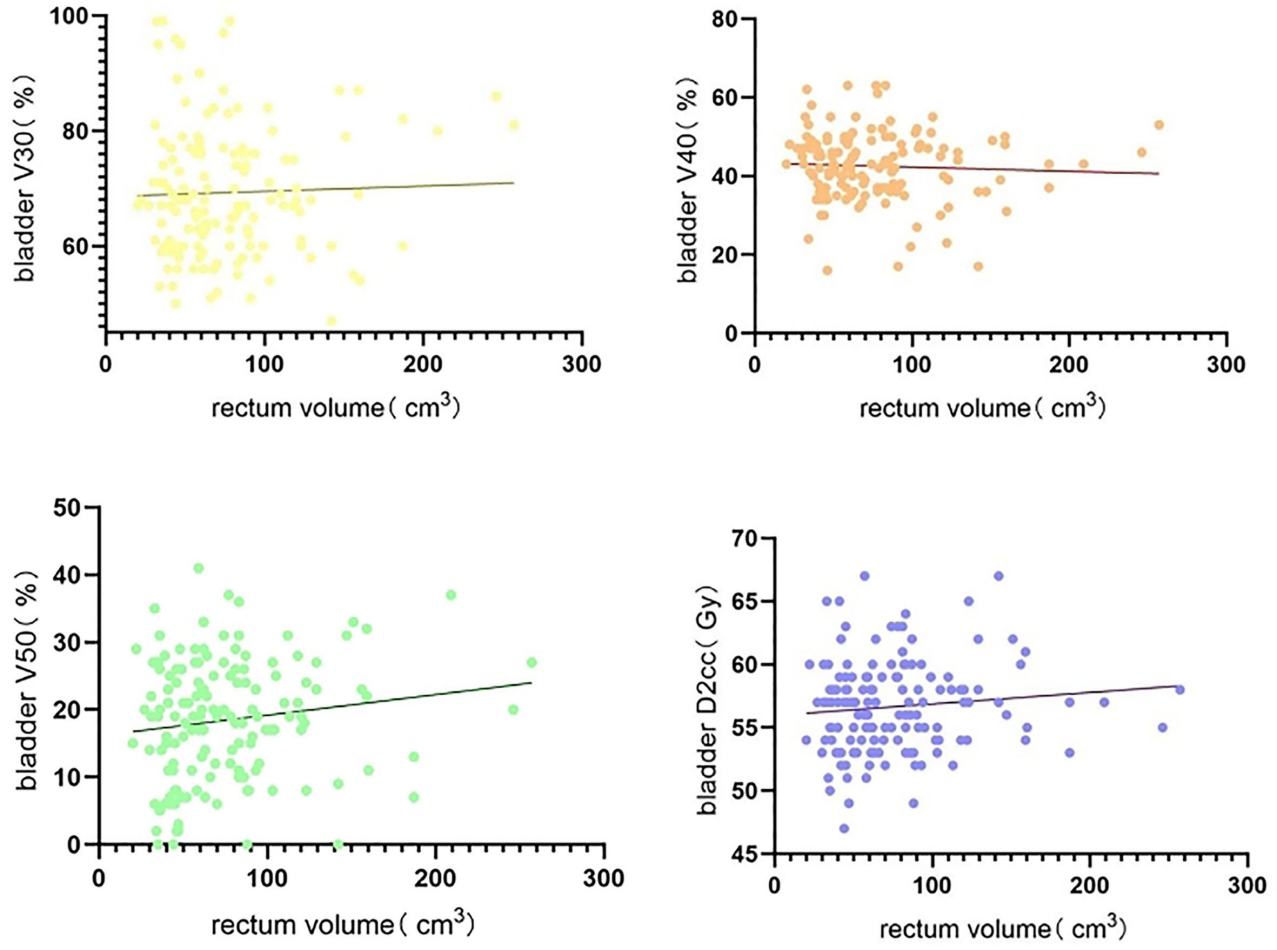
Non-linear regression analysis of increased rectum Volume on bladder V30, V40, V50, D2cc.

**Figure 3:**
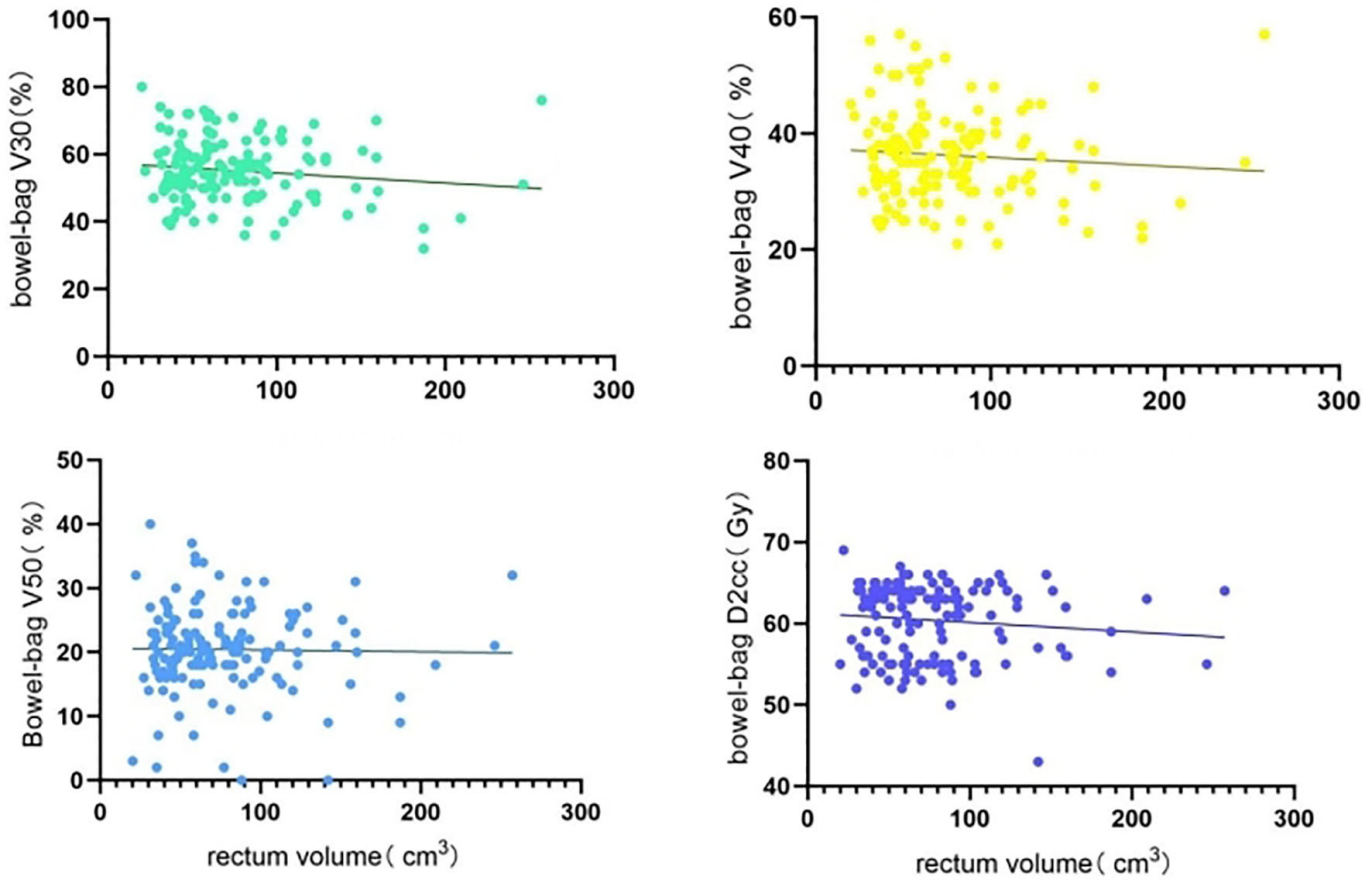
Non-linear regression analysis of increased rectum volume on small bowel V30, V40, V50, D2cc.

Non-linear regression analysis of rectum volume increase on bladder, rectum, and small bowel V30, V40, V50, and D2cc, with rectum volumes ranging from 20 to 257 cm^3^, showed large individual variation across patients with a wide span of rectum volumes. Rectum V30 showed a decreasing trend with increasing rectum volume, whereas rectum V50 and D2cc exhibited a significant increasing trend with increasing rectum volume (**Figure 1**). Bladder V40 showed a decreasing trend with increasing rectum volume, whereas bladder V50 and D2cc exhibited a significant increasing trend with increasing rectum volume (**Figure 2**). Small intestine V30 and V40 showed a decreasing trend with increasing bladder volume, with no significant trend for V50 and D2cc (**Figure 3**).

The 143 patients were divided into three groups according to different rectum volumes: Group A: 21 patients with V<40 ml; Group B: 59 patients with 40 ≤ V < 70 ml; and Group C: 63 patients with V ≥ 70 ml.

**Figure 4:**
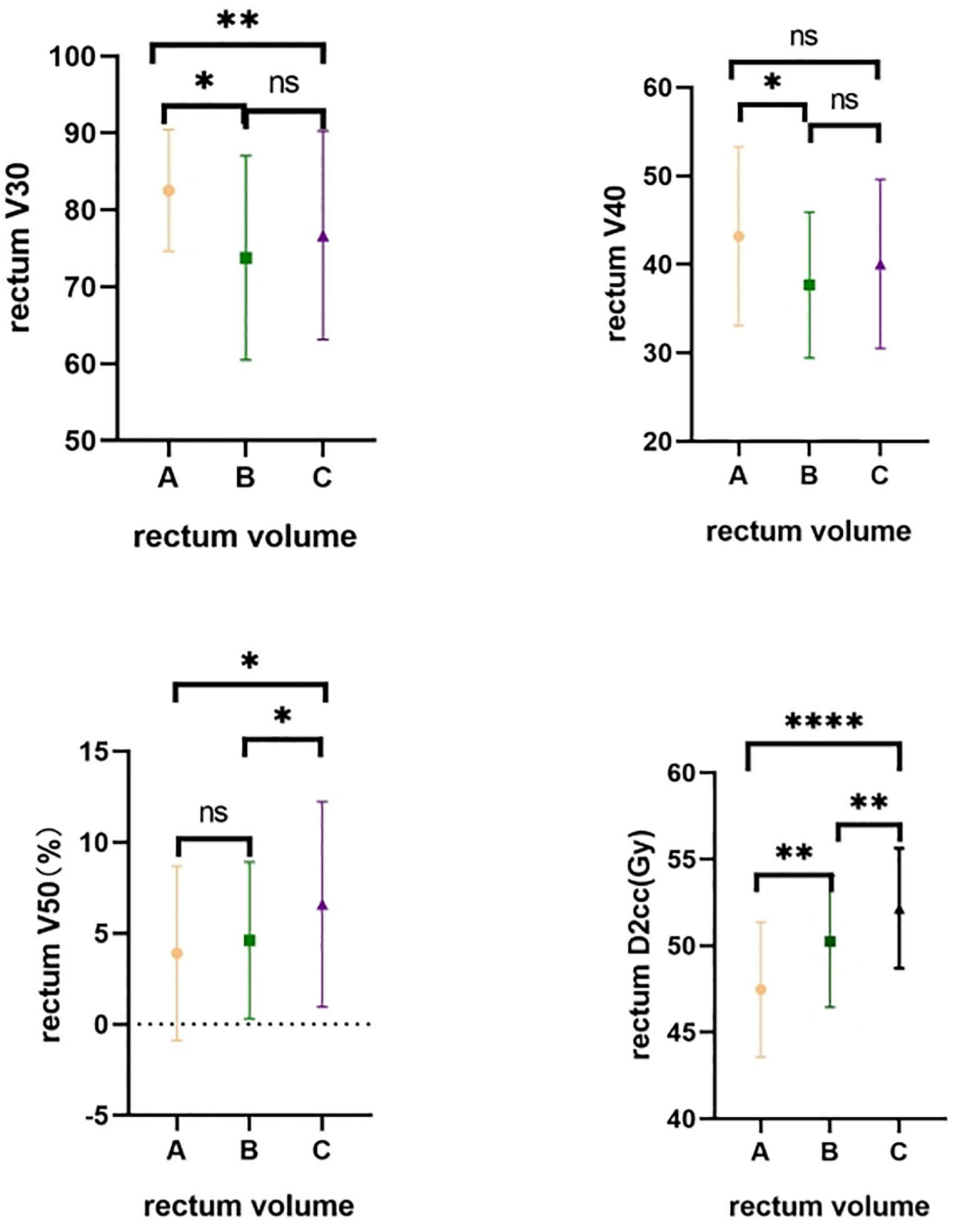
Comparative rectum dose distribution in different groups. A-Group A; B-Group B; C-Group C. ns means no statistical difference; *means P value less than 0.05; **means P value less than 0.01; ***means P value less than 0.001; **** means P value less than 0.0001.

Comparison of the impact of different rectum volumes on the rectum (V30, V40, V50, D2cc) showed significantly higher rectum V30 in Group A than in Groups B and C, and higher rectum V40 in Group A than in Group B (both p < 0.05). Rectum V50 was lower in Group A and Group B than in Group C (p < 0.05). Rectum D2cc was significantly lower in Group A and Group B than in Group C (p < 0.0001) (**Table 2** and **Figure 4**).

**Table 2:** Comparative rectal dose distribution in different groups.

Group	Rectum volume	N	Rectal V30 (%)	Rectal V40 (%)	Rectal V50 (%)	Rectal D2cc (Gy)
Group A	V < 40 ml	21	82.52 ± 7.897	43.19 ± 10.11	3.905 ± 4.795	47.48 ± 3.907
Group B	40 ≤ V < 70 ml	59	73.78 ± 13.30	37.68 ± 8.247	4.610 ± 4.303	50.24 ± 3.798
Group C	V ≥ 70 ml	63	76.70 ± 13.56	40.06 ± 9.548	6.713 ± 5.641	52.16 ± 3.474
Multiple comparisons		P(AB)	0.0007***	0.0155*	0.5331	0.0058**
		P(AC)	0.0195*	0.2038	0.0391*	<0.0001****
		P(BC)	0.2328	0.1435	0.0331*	0.0042**
Overall comparison		P	0.0282*	0.0521	0.0607	<0.0001****

ns means no statistical difference, *means P value less than 0.05, **means P value less than 0.01, ***means P value less than 0.001, **** means P value less than 0.0001.

**Figure 5:**
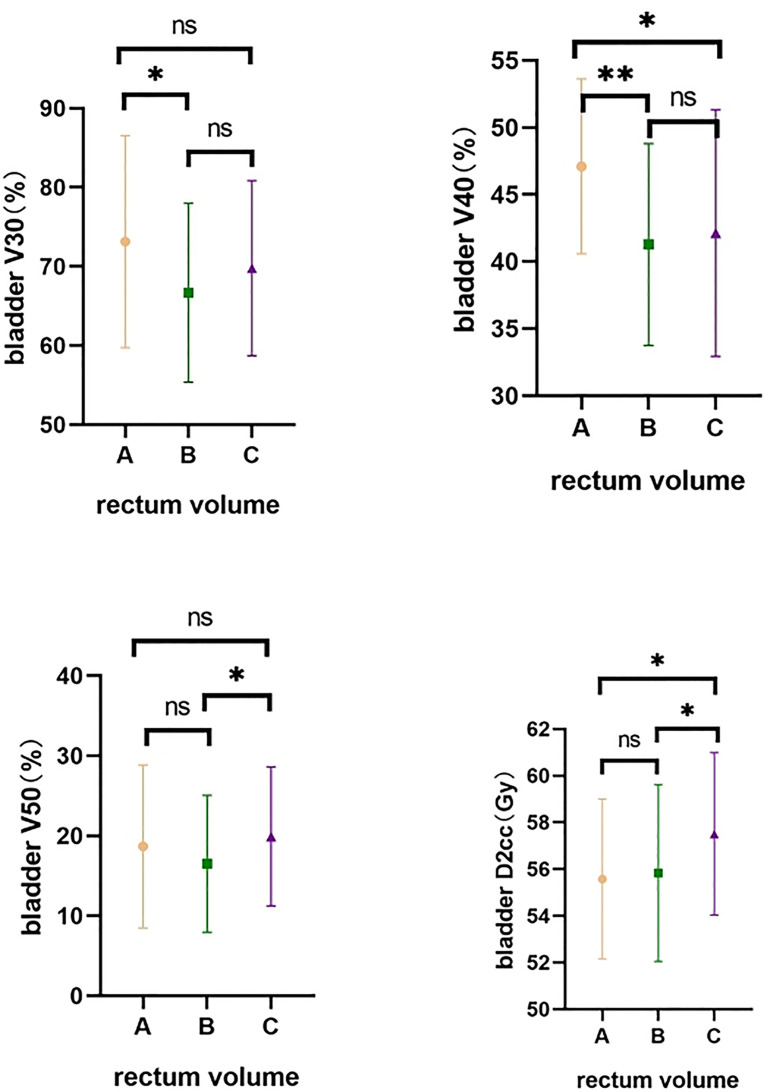
Comparative bladder dose distribution in different groups. A-Group A; B-Group B; C-Group C. ns means no statistical difference, *means P value less than 0.05, **means P value less than 0.01.

The effect of different rectum volumes on the bladder (V30, V40, V50, D2cc) was also compared. The analysis showed higher bladder V30 in Group A than in Group B, and higher bladder V40 in Group A than in Groups B and C (both p < 0.05). Bladder V50 was lower in Group B than in Group C, and bladder D2cc was significantly lower in Group A and Group B than in Group C (both p < 0.05) (**Table 3** and **Figure 5**).

**Table 3:** Comparative bladder dose distribution in different groups.

Group	Rectum volume	N	bladder V30 (%)	bladder V40 (%)	Bladder V50 (%)	Bladder D2cc (Gy)
Group A	V < 40 ml	21	73.14 ± 13.42	47.10 ± 6.526	18.67 ± 10.20	55.57 ± 3.429
Group B	40 ≤ V <70 ml	59	66.69 ± 11.31	41.27 ± 7.524	16.51 ± 8.543	55.83 ± 3.788
Group C	V ≥ 70 ml	63	69.78 ± 11.07	42.13 ± 9.194	19.92 ± 8.687	57.51 ± 3.473
Multiple comparisons		*P*(AB)	0.0360*	0.0023**	0.3481	0.7836
		*P*(AC)	0.2564	0.0247*	0.5851	0.0120*
		*P*(BC)	0.1308	0.5762	0.0308*	0.0292*
Overall comparison		*P*	0.0723	0.0197*	0.1064	0.0172*

*means P value less than 0.05, **means P value less than 0.01.

**Figure 6:**
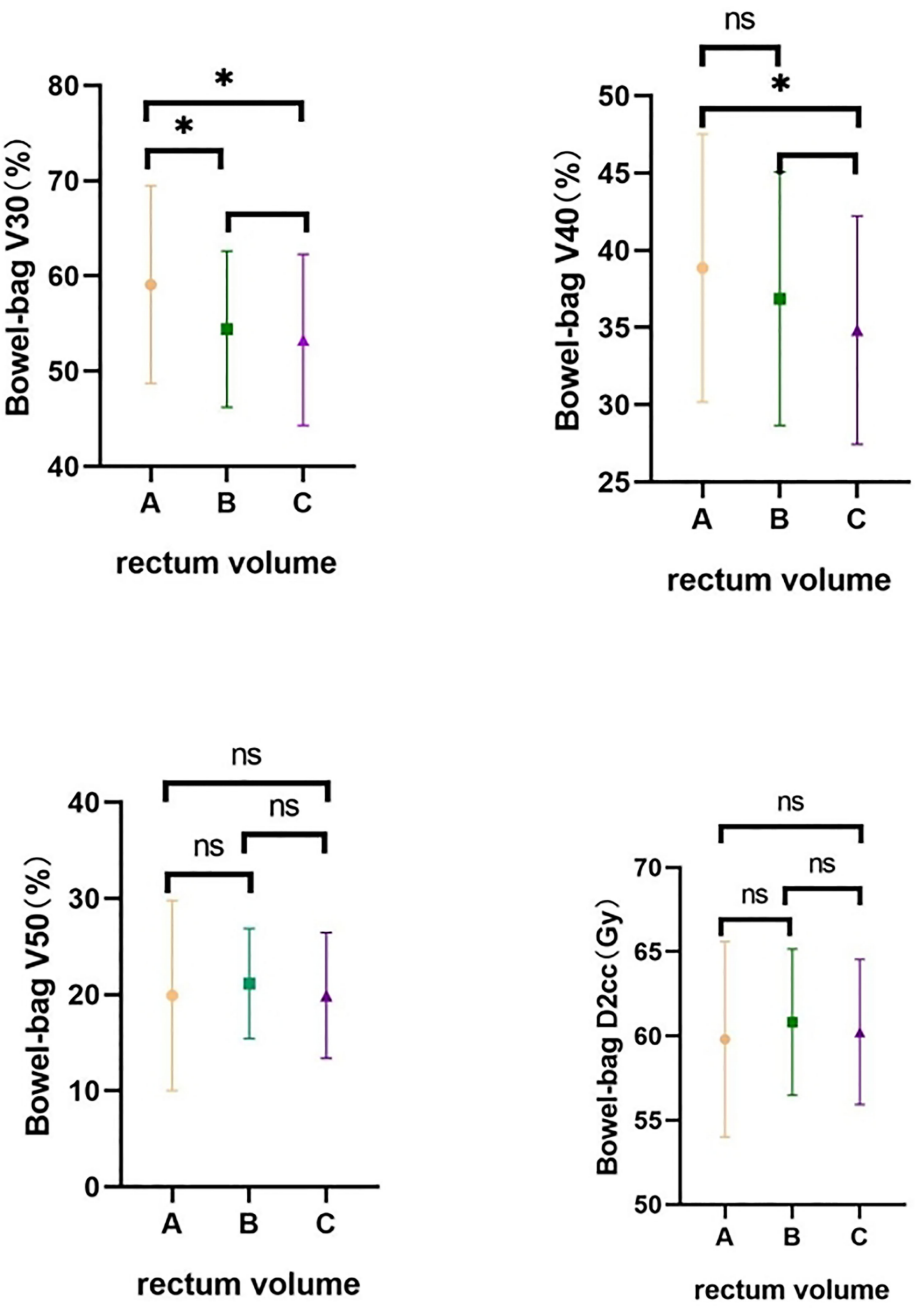
Comparative small bowel dose distribution in different groups. A-Group A; B-Group B; C-Group C. ns means no statistical difference, *means P value less than 0.05.

Small bowel V30 was significantly higher in Group A than in Groups B and C, and higher small bowel V40 in Group A than in Group C (both p < 0.05). There was no difference between the groups for small bowel V50 and D2cc (all p > 0.05) (**Table 4** and **Figure 6**).

**Table 4:** Comparative small bowel dose distribution in different groups.

Group	Rectum volume	N	Small bowel V30 (%)	Small bowel V40 (%)	Small bowel V50 (%)	Small bowel 2cc (Gy)
Group A	V < 40 ml	21	59.10 ± 10.39	38.86 ± 8.673	19.90 ± 9.874	59.81 ± 5.793
Group B	40 ≤ V < 70 ml	59	54.41 ± 8.202	36.86 ± 8.199	21.15 ± 5.726	60.83 ± 4.332
Group C	V ≥ 70 ml	63	53.29 ± 9.004	34.84 ± 7.386	19.92 ± 6.529	60.24 ± 4.302
Multiple comparisons		P(AB)	0.0396*	0.3490	0.4867	0.4002
		P(AC)	0.0159*	0.0421*	0.9933	0.7189
		P(BC)	0.4745	0.1543	0.2714	0.4502
Overall comparison		P	0.0368*	0.1030	0.5665	0.6210

*means P value less than 0.05.

### Analysis of the Risk of Radiation Cystitis and Radiation Proctitis

A total of 10 patients were lost to follow-up, and 10 patients died; 123 were actually followed up. The incidence of radiation cystitis and proctitis was lower in Group B, with a higher incidence of mild cystitis and proctitis in Group A and a higher incidence of mild and moderate or above radiation cystitis and proctitis in Group C (all p < 0.05) (**Table 5**).

**Table 5:** Comparative radiographic cystitis incidence and proctitis incidence distribution in different groups.

Group	N (all)	N (actual)	Mild radiation cystitis	Moderate radiation cystitis	Severe radiation cystitis	p
Group A	21	19	5 (26.32%)	0 (0.00%)	0 (0.00%)	0.047*
Group B	59	53	10 (18.87%)	0 (0.00%)	0 (0.00%)	
Group C	63	51	24 (47.06%)	3 (5.89%)	0 (0.00%)	
Group	N (all)	N (actual)	Radiation proctitis degree I	Radiation proctitis degree II	Radiation proctitis degree III	p
Group A	21	19	3 (15.79%)	1 (5.26%)	0 (0.00%)	0.0395*
Group B	59	53	7 (13.21%)	1 (1.89%)	0 (0.00%)	
Group C	63	51	15 (29.41%)	6 (11.76%)	0 (0.00%)	

*means P value less than 0.05.

## Discussion

Radiotherapy has achieved a satisfying result in the treatment of cervical cancer, especially with the advent of novel technologies, and many studies have proven that new radiotherapy techniques improve target doses while reducing treatment-related toxicity (15–18). However, radiation-induced cystitis and proctitis are still inevitable due to the anatomical position and physical properties of X-rays (10–20). Among the long-term effects reported by patients with cervical cancer, bladder dysfunction and intestinal dysfunction are very common and serious complications, which seriously affect the quality of life of patients (21–23). Therefore, how to reduce the dose of bladder rectum exposure is key to reducing complications. Adjusting the rectum volume to change the dose of OARs may be a feasible and cost-effective approach, but few studies have been done. We have a patent for a rectal balloon used in prostate cancer radiotherapy, patent number: CN 201320826810, which can fix the position and volume of rectum. Therefore, we conducted this study to find the right rectal volume for external irradiation of cervical cancer.

Our study indicates that during external irradiation for cervical cancer, rectum volumes of 40–70 ml have the least side effects on the bladder, rectum, and small intestine. When the volume of the rectum becomes larger during external irradiation for cervical cancer, the volume of the rectum away from the cervical lesion increases, resulting in higher V30 and V40 in the rectum when the rectum is less than 40 ml. However, as the rectal volume increases, the rectal wall in the area of the cervical lesion is closer to the cervical lesion, resulting in greater V50 and D2cc of the rectum when the rectal volume is greater than 70 ml. With the increase in rectal volume, the position of the cervix and bladder moved forward and the bladder as a whole was relatively far from the high-dose area of the cervical lesion, resulting in higher V30 and V40 in the bladder when the rectal volume was less than 40 ml. However, as the rectal volume increases, the bladder wall in the cervical lesion area is closer to the cervical lesion, resulting in greater V50 and D2cc in the bladder for rectal volumes greater than 70 ml. With increased rectal volume, the overall position of the small intestine shifts upward, resulting in higher V30 and V40 in the small intestine when the rectum is less than 40 ml.

In contrast, a rectum volume of less than 40 ml increased V30 and V40 in the rectum bladder as well as in the small intestine and increased the incidence of mild radiation cystitis and mild radiation proctitis, considering that the incidence of mild radiation cystitis and mild radiation proctitis is more closely related to the volume of the low-dose area of the rectum bladder and small intestine. A rectum volume greater than 70 ml increases V50 and D2cc in the rectum and bladder and increased the incidence of more than moderate radiation cystitis and radiation proctitis, considering that the incidence of more than moderate radiation cystitis and radiation proctitis is related to the maximum dose point received by the bladder and rectum.

Because rectal volume is difficult to control, we often ask patients to empty their rectum before treatment in clinical practice. However, even if the patient is asked to empty the rectum, it is still not possible to accurately control the rectal volume. We have a patent for a rectal balloon used in prostate cancer radiotherapy, patent number: CN 201320826810, which can fix the position and volume of the rectum. Following this study, we will use the patent for subsequent clinical trials and believe that strict control of the patient’s rectal volume and position will further reduce side effects regarding the rectum and bladder after radiation therapy for patients with cervical cancer.

Several limitations need to be mentioned. First, this was a retrospective study, and the sample size was relatively small; a well-designed prospective cohort with a large sample size of patients is needed. Second, changes in bladder volume may affect experimental data. If the bladder volume can be kept consistent, the results of the study will be more reliable. Third, during treatment, it is difficult to keep the rectum volume consistent with the positioning of the patient. If rectum consistency can be guaranteed by daily image guidance, the conclusion will be more convincing.

## Conclusion

For external irradiation in patients with cervical cancer, a rectum volume of 40–70 ml seems most appropriate, whereas >70 ml increases the risk of severe radiation cystitis and radiation proctitis, and <40 ml increases the risk of mild radiation cystitis and mild radiation proctitis.

## Data Availability Statement

The original contributions presented in the study are included in the article/supplementary material. Further inquiries can be directed to the corresponding author.

## Ethics Statement

The studies involving human participants were reviewed and approved by the Ethics Committee of the Second Hospital of Hebei Medical University. Written informed consent to participate in this study was provided by the patients/participants.

## Author Contributions

YJW—study design, data analysis, writing, and final corrections. CL—data gathering. WW—data gathering, data analysis. LT—data gathering. ZX—data analysis, writing of the initial drafts. YQW—data examination. HG—data gathering. XX—study design and final corrections. All authors contributed to the article and approved the submitted version.

## Conflict of Interest

The authors declare that the research was conducted in the absence of any commercial or financial relationships that could be construed as a potential conflict of interest.

## Publisher’s Note

All claims expressed in this article are solely those of the authors and do not necessarily represent those of their affiliated organizations, or those of the publisher, the editors and the reviewers. Any product that may be evaluated in this article, or claim that may be made by its manufacturer, is not guaranteed or endorsed by the publisher.
